# Quantifying DNA damage on paper sensors *via* controlled template-independent DNA polymerization†

**DOI:** 10.1039/d1sc04268h

**Published:** 2022-04-20

**Authors:** Wei Xue, Qiang Zhang, Yangyang Chang, John D. Brennan, Yingfu Li, Meng Liu

**Affiliations:** School of Environmental Science and Technology, Key Laboratory of Industrial Ecology and Environmental Engineering, Ministry of Education, Dalian University of Technology Dalian 116024 China mliu@dlut.edu.cn; School of Bioengineering, Dalian University of Technology Dalian 116024 China; Dalian POCT Laboratory Dalian 116024 China; Biointerfaces Institute, McMaster University 1280 Main Street West Hamilton Ontario L8S4O3 Canada brennanj@mcmaster.ca; Department of Biochemistry and Biomedical Sciences, McMaster University 1280 Main Street West Hamilton Ontario L8S4K1 Canada

## Abstract

We report on a paper-based sensor capable of performing template-independent DNA synthesis by terminal deoxynucleotidyl transferase (TdT). Importantly, we observed that TdT efficiently incorporates fluorescently labeled dUTP on to 3′-OH ends of DNA strands in a strictly controllable manner on cellulose paper, in comparison to its distributive mode of DNA synthesis in solution. Due to the high roughness and porous nature of cellulose paper, we attribute this controllable DNA polymerization to the pore confinement effect on the catalytic behaviour of TdT. Taking advantage of this finding, we proposed a paper-assisted TdT (PAT) assay for absolute quantification of alkylated DNA lesions (N7-methylguanine), DNA deamination (cytosine-to-uracil) and DNA oxidation (8-oxo-7,8-dihydroguanine) by combining various DNA glycosylases. This PAT assay provides a low-cost, high throughput and easy to use method for quantifying the absolute levels of various types of DNA lesions, thus making it well-suited for drug development, genotoxicity testing, and environmental toxicology.

## Abstract 2

Terminal deoxynucleotidyl transferase (TdT) catalyzes template-independent DNA synthesis in a well-controllable mode on paper, allowing absolute quantification of polymetric labeling of a single 3′-OH present on genomic DNA.

## Graphical abstract

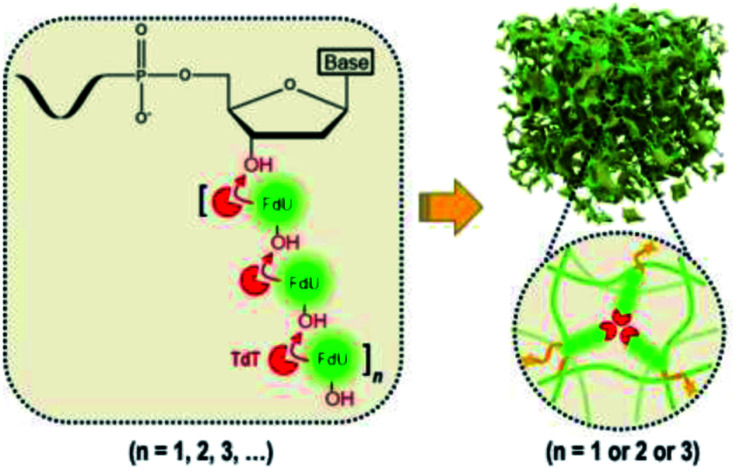

## Introduction

Paper, or cellulose in general, has recently been regarded as an ideal platform for engineering simple and low-cost analytical devices in the fields of clinical diagnostics, food safety and environmental monitoring.^1^ To date, paper-based sensors have been widely used for nucleic acid (DNA or RNA) detection.^2–6^ A common approach for achieving sensitive target detection involves the use of isothermal nucleic acid amplification (INAA) methods, such as rolling circle amplification (RCA),^2^ loop-mediated isothermal amplification (LAMP),^3^ recombinase polymerase amplification (RPA),^4^ helicase dependent amplification (HDA),^5^ and nucleic acid sequence based amplification (NASBA).^6^ All these existing INAA methods use template-dependent polymerases that catalyze the incorporation of mononucleotides into a short primer annealed to a DNA or RNA template. However, these polymerases may not be useful for amplifying damaged DNA or RNA simply because the target of concern may contain strand breaks.

DNA damage induced by endogenous and exogenous chemical agents plays a critical role in various biological processes such as mutagenesis, carcinogenesis and aging in humans.^7^ Consequently, detection and characterization of DNA damage and repair is essential for evaluating their biological impact.^8^ dUTP nick end labeling with terminal deoxynucleotidyl transferase (TdT), also known as the TUNEL assay, has been commonly employed to detect a range of DNA damage *in vivo* and *in vitro*.^9^ The principle of this assay is based on the ability of TdT, a unique DNA template-independent polymerase, to incorporate fluorescently-labeled dUTP onto 3′-OH ends of DNA strand breaks. However, this assay is intrinsically unquantifiable due to the distributive mode of DNA labeling by TdT, thus rendering it unable to directly measure the numbers of DNA breaks.^10^ Currently, using 2′,3′-ddUTP can ensure that one labeled ddU is added to each 3′-OH DNA end, allowing the absolute quantification of generated fluorescence signals.^10^ However, a longer reaction time (up to 24 hours) is required for TUNEL reaction due to the decreased catalytic activity of TdT on 2′,3′-ddUTP.

Herein, we describe for the first time the use of TdT to perform controlled DNA synthesis on paper. In particular, we report on the intriguing finding that TdT catalyzes the non-templated addition of nucleotides to 3′-OH ends of DNA initiators with a well-controlled degree of polymerization (DP) on paper relative to solution, thus enabling the quantifiable polymeric labeling of a single 3′-OH end. We further demonstrate a paper-assisted TdT (denoted as PAT) assay that can be used for absolute quantification of the levels of methylated DNA lesions (N7-methylguanine), DNA deamination (cytosine-to-uracil), and DNA oxidation (8-oxo-7,8-dihydroguanine) on genomic DNA.

## Results and discussion

### TdT polymerization in solution and on paper

**Fig. 1:**
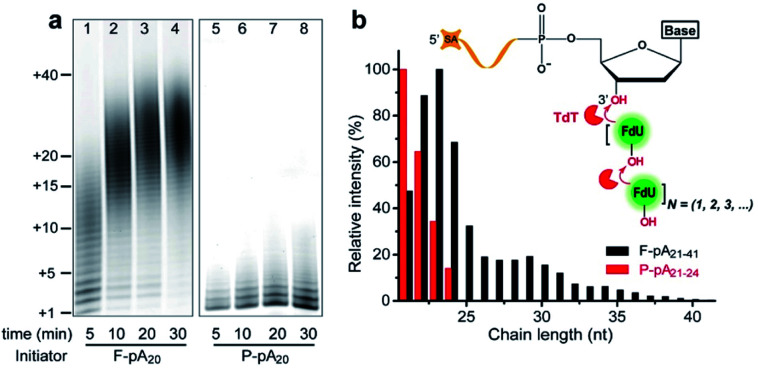
(a) TdT elongation of pA_20_ initiator. Reactions were carried out with 20 μM FdU, 0.5 μM pA_20_ and 35 nM TdT at room temperature for various reaction times. Aliquots of the reactions taken at 5, 10, 20, and 30 min were analyzed on a 20% dPAGE (8 M urea) gel. (b) Size distribution of the major products at 5 min. The inset shows that each incorporated FdU generates a new available FdU 3′-OH end for the subsequent incorporation by TdT. SA: streptavidin.

We first carried out TdT enzymatic assays in solution. Free poly(adenine) initiator of 20 nt (F-pA_20_, see Table S1† for all DNA molecules used in this study) was incubated with TdT and FITC-labeled dUTP (FdU) to allow the incorporation of fluorophore into the products to aid in identification by denaturing polyacrylamide gel electrophoresis (dPAGE) (Fig. 1a, lanes 1–4).

Note that the marker indicates the lengths of the products, which was confirmed by dPAGE analysis of chain length distribution using different DNA ladders (Fig. S1†). Typical sizes of the major products ranged from 21 nt to 41 nt at 5 min (lane 1), indicating that the population of polymers is heterogeneous (Fig. 1b, black column). Furthermore, TdT was able to synthesize long polymers over time, with more than 40 FdU molecules incorporated onto a single 3′-OH end at 30 min (lane 4). This result is due to the fact that each incorporated FdU creates a new 3′-OH end on pA_20_ for subsequent polymerization (Fig. 1b, inset). Thus, TdT catalyzes the DNA synthesis in a distributive mode in solution.

We then performed the TdT polymerization reaction on paper (Fig. 1a, lanes 5–8). We first used the wax-printing technique to print hydrophobic wax barriers on a Whatman Grade 1 paper plate, with the diameter of each well being 4 mm (Fig. S2†). pA_20_ initiators were first immobilized *via* the adsorption of streptavidin bound with biotinylated pA_20_ (see ESI† for details). A retention efficiency of 85 ± 3% was obtained for paper-bound pA_20_ (P-pA_20_) (Fig. S3†). After incubating P-pA_20_ with a mixture of TdT, FdU and reaction buffer, the elongation of P-pA_20_ was also observed at 5 min (lane 5). However, the polymer population was homogeneous (Fig. 1b, red column). The major products were labeled with only 1 to 4 FdU even after 30 min (Fig. 1a, lane 8). Thus, the mode of polymerization of P-pA_20_ by TdT is controllable on cellulose paper.

### Controllable TdT polymerization on paper

**Fig. 2:**
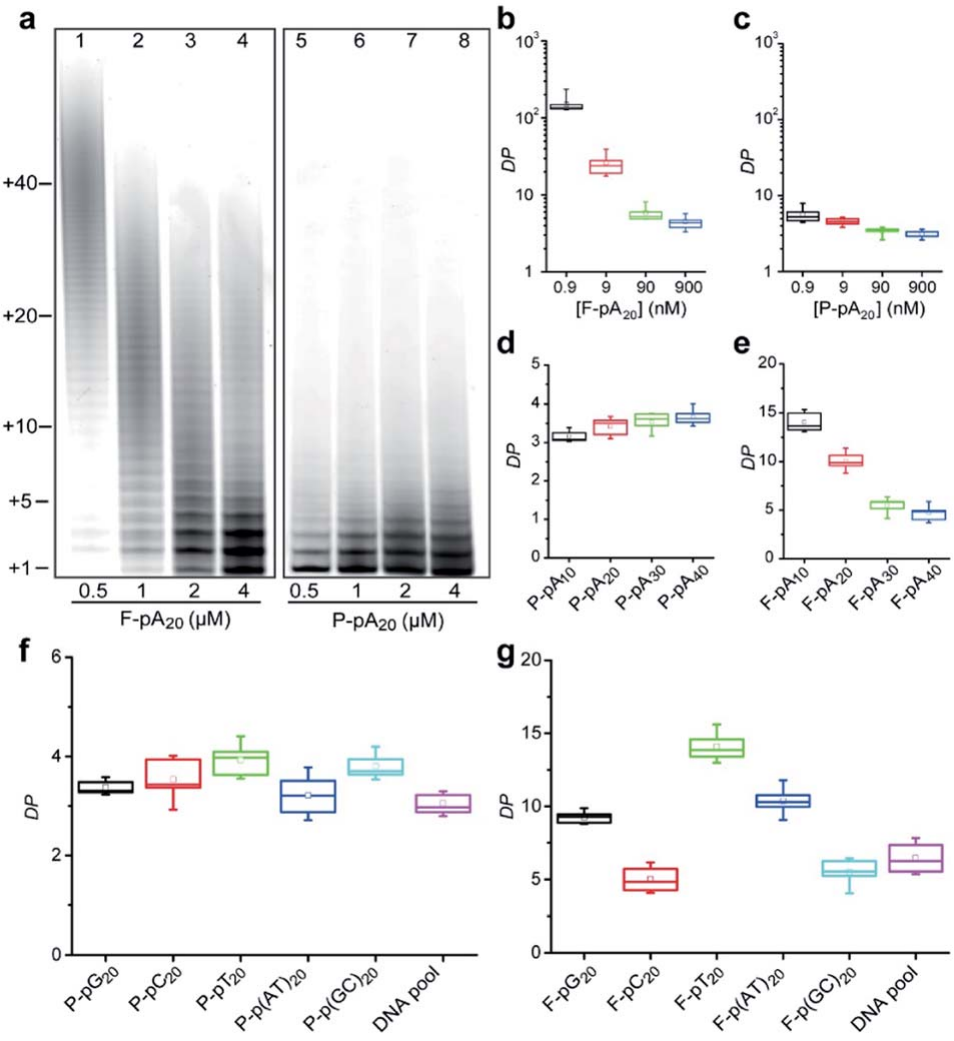
(a) dPAGE analysis of the products synthesized by TdT at various concentrations of pA_20_. 35 nM TdT was incubated with 20 μM FdU and pA_20_ as indicated. Aliquots of the reaction were taken at 30 min. Effect of (b) F-pA_20_ and (c) P-pA_20_ concentration on the average DP. Effect of the size of (d) P-pA and (e) F-pA on the average DP. Effect of the sequence of the DNA initiator on the average DP for TdT polymerization (f) on cellulose paper and (g) in solution.

We also studied the degree of processivity of TdT at various initiator concentrations. As shown in Fig. 2a, there is a slight reduction in the size of the products when the F-pA_20_ concentration is increased, but the chain length distribution covers a broad range (lanes 1–4). For paper-based TdT elongation, the population of polymers remains uniform even at high P-pA_20_ concentrations (lanes 5–8). Typical sizes of the major products ranged from 21 nt to 24 nt. For reproducibility, <12% coefficient of variation was observed when comparing between the products (Table S2†). By determining the total amounts of the incorporated FdU on paper (Fig. S4†) and the elongated pA_20_ by dPAGE (Fig. S5†), we can estimate the average DP of the product (see ESI† for details). For the solution-based strategy, the average DP decreased from 149.8 ± 34.9 (mean ± standard deviation) to 4.5 ± 0.8 (33-fold) with increasing pA_20_ concentrations from 0.9 nM to 900 nM (Fig. 2b). However, the average DP only changed from 5.6 ± 1.4 to 3.1 ± 0.4 (1.8-fold) for the paper-based strategy when the pA_20_ concentration increased by 10^3^-fold (Fig. 2c). Furthermore, the obtained DP was independent of the TdT concentration (Fig. S6†), which is different from that of regular solution-based strategy.

We next examined if this controllable synthesis by TdT required a specific size of DNA initiator (Fig. S7†). The DP calculated for P-pA_10_, P-pA_20_, P-pA_30_, and P-pA_40_ was 3.1 ± 0.1, 3.1 ± 0.4, 3.6 ± 0.2, and 3.7 ± 0.3, respectively (Fig. 2d), indicating the observed controllable synthesis is not restricted to a given size of initiator for paper-based strategy. However, the average DP for F-pA_10_, F-pA_20_, F-pA_30_, and F-pA_40_ was more variable, being 14.1 ± 1.0, 10.0 ± 0.9, 5.5 ± 0.8, and 4.7 ± 0.9 (Fig. 2e), respectively, indicating that the size of initiator does affect on the solution-based TdT polymerization to some degree.

To rule out the possibility that the observed controllable mode of DNA synthesis by TdT might be dependent on the sequence of a DNA initiator, we tested other initiators including: poly(guanine) (pG_20_); poly(cytosine) (pC_20_); poly(thymine) (pT_20_); pA_20_–pT_20_; pC_20_–pG_20_; and, a DNA pool (made of a random sequence of 40 nucleotides). The average DP was found to be 3.4 ± 0.1, 3.5 ± 0.4, 3.9 ± 0.3, 3.2 ± 0.4, 3.8 ± 0.3, and 3.1 ± 0.2 for P-pG_20_, P-pC_20_, P-pT_20_, P-p(AT)_20_, P-p(GC)_20_ and the DNA pool (Fig. 2f and S8†), respectively. For comparison, the results of solution-based TdT elongation demonstrated that the obtained DP was highly dependent on the specific initiator sequence (Fig. 2g).

Taken together, these results suggest that the concentration, size or sequence of the DNA initiator as well as the TdT concentration can significantly affect the degree of processivity of TdT in solution, making it impossible to quantify the levels of different types of genomic DNA damage owing to the distributive mode of solution-based TdT polymerization. In sharp contrast, TdT catalyzes the DNA synthesis in a highly controllable manner on cellulose paper. Thus, integrating TdT-based polymerization with paper sensors should lead to the emergence of a new assay platform that allows the absolute levels of different types of DNA damage to be quantified.

We also compared the activities of TdT on F-pA_20_ and P-pA_20_ initiators. TdT is able to incorporate 2.7 pmol FdU into F-pA_20_ in 120 min, whereas it elongates P-pA_20_ within 30 min, and reaches a plateau after the incorporation of 1.7 pmol FdU (Fig. S9†). Hence, the initial incorporation rate was increased 5-fold from 0.06 min^−1^ for F-pA_20_ to 0.32 min^−1^ for P-pA_20_. This result highlights an important advantage of paper-assisted TdT polymerization in terms of reaction kinetics.

### Pore confinement on TdT polymerization

**Fig. 3:**
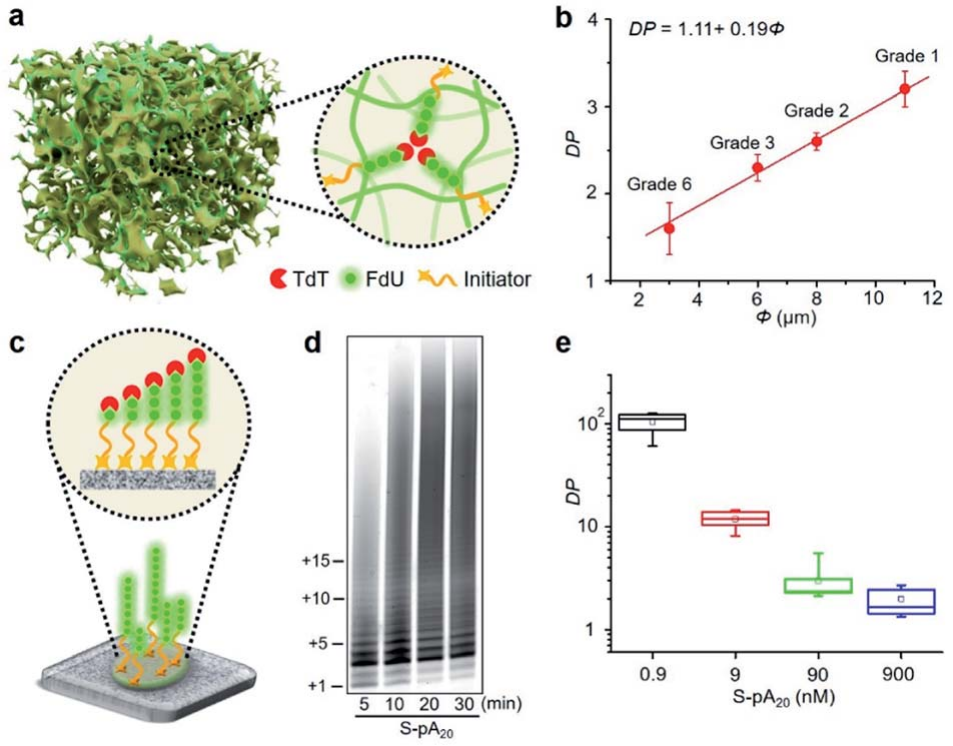
(a) Schematic representation of paper-based TdT polymerization in the confined environment provided by the pores of cellulose paper. (b) Average DP plotted against pore size (*Φ*) of different cellulose papers. (c) TdT elongation of S-pA_20_ on the surface of nitrocellulose membrane (Millipore HF120). (d) dPAGE analysis of the elongated S-pA_20_. Reactions were carried out with 20 μM FdU, 50 nM S-pA_20_ and 35 nM TdT at room temperature for various times. (e) Effect of S-pA_20_ concentration on the average DP.

Since cellulose paper is known for its ordered network and porous structure,^11^ we hypothesized that the accessibility of elongated DNA initiators to the active site of a TdT may be reduced in a molecularly crowded environment. Hence, TdT will dissociate from an extended initiator to re-initiate DNA synthesis on a shorter, more accessible one, thus improving the homogeneity of the final polymer population (Fig. 3a). We carried out two experiments to confirm this hypothesis. The first experiment examined the effect of pore size (*Φ*) on the average DP for P-pA_20_ (Fig. S10†). As shown in Fig. 3b, decreasing *Φ* (from 11 μm to 3 μm) resulted in a decrease in the DP (from 3.2 ± 0.2 to 1.6 ± 0.3) in a linear fashion (DP = 1.11 + 0.19*Φ*). The second experiment involved the use of surface-bound biotinylated pA_20_ (S-pA_20_), attached to streptavidin-coated nitrocellulose membrane (Fig. 3c). In this case, the accessibility of S-pA20 to TdT was not reduced. As shown in Fig. 3d, the size of the products grew over time. The average DP decreased from 106 ± 10 to 2.1 ± 0.5 with increasing S-pA_20_ concentrations from 0.9 nM to 900 nM (Fig. 3e and S11†), reflecting the distributive mechanism of TdT polymerization on the membrane surfaces. Taken together, these results confirm the effect of pore confinement on controlling the catalytic behavior of TdT onto the pores of cellulose paper.

### Quantification of DNA alkylation damage

**Fig. 4:**
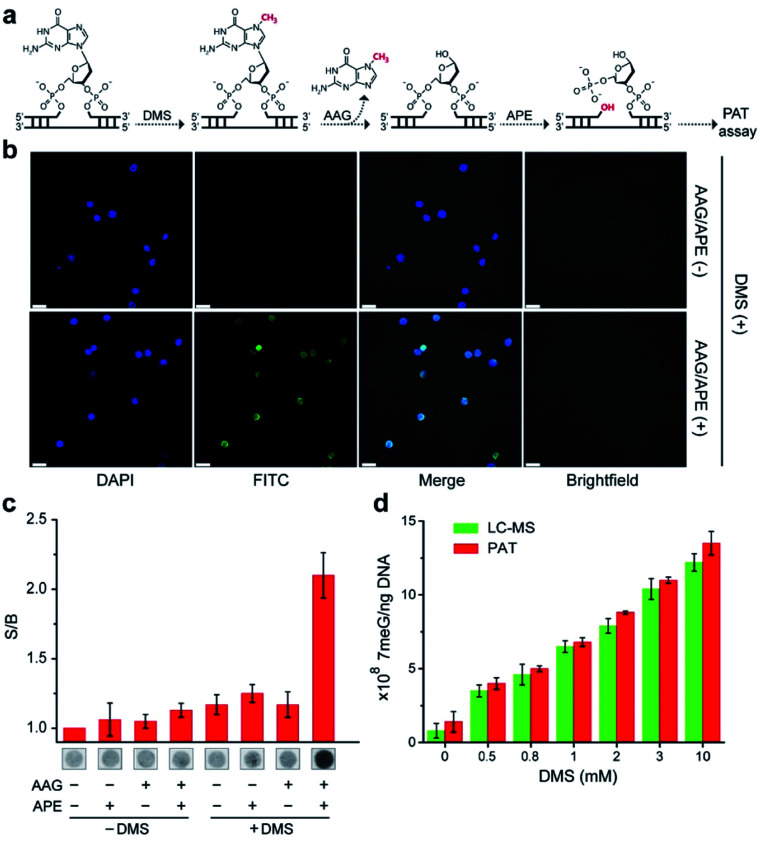
(a) Strategy of FdU labeling of 7meG lesions by AAG/APE-mediated PAT assay. The DMS-induced damaged base, 7meG is first removed by AAG, and APE cleaves the abasic site, generating 3′-OH ends for subsequent FdU labeling by TdT on paper. (b) *In situ* labeling of 7meG in DMS-treated ZFL cells. Scale bar: 10 μm. (c) S/B values for paper sensors carried out under different reaction conditions. The extracted genomic DNA was mixed with AAG/APE at 37 °C for 60 min, followed by incubation with TdT and FdU on paper at room temperature for 30 min. The others represent various controls. (d) Absolute quantification of 7meG by mass spectrometric method and PAT assay. ZFL cells (1 × 10^7^) were first treated with varying concentrations of DMS for 30 min before lysis. The error bars in (c) and (d) represent standard deviations of three independent experiments.

We next examined the possibility of exploiting the PAT assay for quantification of the absolute levels of DNA alkylation damage, a common type of DNA lesion induced by alkylating agents.^12^ We first treated the zebrafish liver (ZFL) cells with dimethyl sulfate (DMS) to generate a high fraction of N7-methylguanine (7meG) lesions on genomic DNA. The corresponding cellular viability was determined to be above 80% after DMS treatment (Fig. S12†). Upon exposure to alkyladenine DNA glycosylase (AAG) and AP-endonuclease (APE), which are commonly used in base excision repair (BER),^13^ these 7meG sites can be converted into 3′-OH ends (Fig. 4a). As expected, the genomic DNA was resolved on a gel yielding smeared bands with a broad size range (Fig. S13†). TdT catalyzes the addition of FdU onto these 3′-OH ends in ZFL cells. *In situ* results using an AAG/APE-mediated TUNEL assay validate this approach (Fig. 4b and S14†).

We then carried out the TdT polymerization on a paper sensor. It has demonstrated that cellulose paper can be used for DNA extraction from biological samples due to the physical entanglement of genomic DNA with the fiber matrix.^14^ Therefore, the genomic DNA should be easily entrapped into paper without using biotin modification. To confirm this, we first evaluated the DNA capture efficiency of two Whatman cellulose papers (Grade 1 and Grade 6). The polymerase chain reaction (PCR) results indicated that the Grade 1 paper provided a capture efficiency of 89 ± 3% for unmodified genomic DNA (Fig. S15†). Following polymerization on paper, Fig. 4c shows that a large fluorescence signaling magnitude (*i.e.*, S/B, defined as the fluorescence intensity in the presence of DNA over that in the absence of any target) was generated, when: (1) ZFL cells were first treated with DMS; (2) the extracted genomic DNA was treated with AAG/APE. Assuming the average DP = 3 for Grade 1 paper (Fig. 3b), this means that only three FdU molecules are added to each 3′-OH DNA end by TdT. Thus, we determined the levels of 7meG to be (1.4 ± 0.7) × 10^8^, (4 ± 0.4) × 10^8^, (5 ± 0.2) × 10^8^, (6.8 ± 0.3) × 10^8^, (8.8 ± 0.1) × 10^8^, (11 ± 0.2) × 10^8^ and (13.5 ± 0.8) × 10^8^ ng^−1^ DNA after exposure to DMS at concentrations of 0, 0.5, 0.8, 1, 2, 3, and 10 mM, respectively (Fig. 4d). This PAT assay provided a detection limit of 10^10^ 7meG molecules (Fig. S16†), on the basis of the 3*σ*/slope (*σ*, standard deviation of the blank samples). As a control, we also quantified the 7meG for the same cell sample set using mass spectrometry (Fig. S17†). Both the mass spectrometric method and our assay produced comparable 7meG levels. Also of note, the PAT assay consistently produced somewhat higher 7meG levels than those from the mass spectrometry. A likely explanation is that DMS treatment can also generate N3-methyladenine (3meA) lesions,^10^ which can also be cleaved by AAG/APE. Future experiments will use alkylguanine DNA glycosylase (AGG) to produce specific lesions. When comparing between 30 individual paper sensors, a coefficient of variation of 5.8% was obtained (Fig. S18†), indicating the good reproducibility of this assay.

### Quantification of other modified bases

**Fig. 5:**
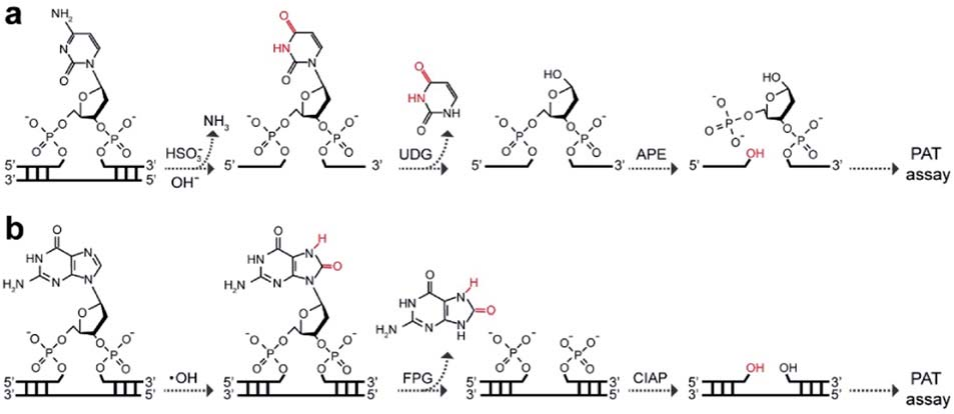
Working principle of (a) UDG/APE- and (b) FPG/CIAP-mediated PAT assay to measure cytosine damages and 8-oxoG sites on genomic DNA, respectively.

To extend the PAT assay beyond DNA alkylation detection, we applied the same strategy for two common forms of DNA damage, including deamination (*e.g.*, cytosine-to-uracil)^15^ and oxidation (*e.g.*, 8-oxo-7,8-dihydroguanine, 8-oxoG).^16^ In the first experiment, we employed an uracil DNA glycosylase (UDG)/APE-mediated PAT assay to measure the absolute amounts of cytosine damage (Fig. 5a). A known amount of uracil-containing pA_19_ target was first tested. Results show good correlation between input and recovered targets with recovery yields between 81% and 99% (Fig. S19†). We then treated genomic DNA with sodium bisulfite to deaminate cytosine into uracil. It was determined that the contents of uracil were (6.2 ± 1.4) × 10^7^, (4.1 ± 0.2) × 10^8^ and (1.6 ± 0.2) × 10^9^ ng^−1^ DNA at bisulfite concentrations of 62.5, 250, and 1000 mM, which is comparable with the values obtained using mass spectrometry (Fig. S20†).

In the second experiment, we treated the genomic DNA with Fenton reagents (H_2_O_2_/Fe^2+^) to generate 8-oxoG lesions (Fig. S21†). Following exposure to formamidopyrimidine-DNA glycosylase (FPG) and calf intestinal alkaline phosphatase (CIAP), we carried out the PAT assay (Fig. 5b). The absolute levels of 8-oxoG were determined to be (4.0 ± 1.9) × 10^7^, (2.6 ± 0.5) × 10^8^ and (4.6 ± 0.2) × 10^8^ ng^−1^ DNA at H_2_O_2_/Fe^2+^ concentrations of 1.6/0.3 mM, 6.4/1.2 mM, 25.6/4.8 mM. Furthermore, we found high levels of 3′-OH ends in these H_2_O_2_/Fe^2+^-treated samples, which is not available for mass spectrometry (Table 1). These results suggested that Fenton chemistry could result in both 3′-OH ends and 8-oxoG sites in genomic DNA. Therefore, our PAT assay has the ability to simultaneously measure multiple DNA damage levels.

**Table 1:** Analysis of 8-oxoG sites and 3′-OH ends in H_2_O_2_/Fe^2+^-treated genomic DNA

H_2_O_2_ (mM)/Fe^2+^ (mM)	PAT assay		Mass spectrometry	
	8-oxoG	3′-OH	8-oxoG	3′-OH
0/0	N.D.^a^	N.D.	N.D.	N.D.
1.6/0.3	(4.0 ± 1.9) × 10^7^	(4.7 ± 0.9) × 10^7^	(1.5 ± 1.0) × 10^7^	N.A.^b^
6.4/1.2	(2.6 ± 0.5) × 10^8^	(1.0 ± 0.4) × 10^8^	(2.7 ± 0.1) × 10^8^	N.A.
25.6/4.8	(4.6 ± 0.2) × 10^8^	(1.3 ± 0.3) × 10^8^	(4.8 ± 0.2) × 10^8^	N.A.

a N.D. = not detected.

b N.A. = not available. Data are averages ± SD.

### Quantification of DNA repair

**Fig. 6:**
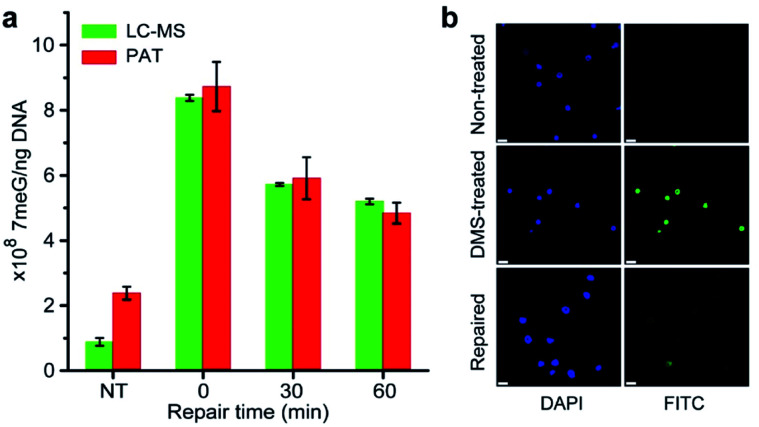
(a) DNA repair kinetics. Non-treated (NT) cells were not exposed to DMS. ZFL cells (1× 10^7^) are treated with 2 mM DMS for 30 min at 0 °C, and allowed to repair at DMEM with 10% FBS at 37 °C for 0, 30, and 60 min before lysis. Error bars represent the standard deviation of the replicates. (b) *In situ* labeling of 7meG in ZFL cells using the AAG/APE-mediated TUNEL assay. DMS-treated cells were cultured at DMEM with 10% FBS at 37 °C for 60 min before labeling. Scale bar: 10 μm.

It is well-known that alkylating agents are the most commonly prescribed chemotherapeutic drugs for cancer chemotherapy.^10^ However, cells have evolved multiple repair mechanisms to counteract the effects of these anticancer drugs.^13^ Thus, the measurement of DNA repair capacity of cells is critical in cancer treatment and drug development. As a proof of concept, we also performed the PAT assay to evaluate the DNA repair capacity by measuring the 7meG levels in DMS-treated cells. As shown in Fig. 6a, nearly 45% of alkylated DNA lesions were repaired within 60 min. This was further confirmed using the AAG/APE-mediated TUNEL assay (Fig. 6b). These results suggest that the PAT assay has the ability to measure the extent of DNA repair.

## Conclusions

Overall, our work demonstrates that TdT-mediated template-independent DNA synthesis can be performed on cellulose paper in a controllable manner. This work indicates that the accessibility of the elongated DNA initiator to TdT can be restricted by physical constraints due to the porous nature of paper materials. Through the incorporation of various DNA glycosylases that selectively recognize and remove the damaged base in base excision repair, we propose a PAT assay to measure the absolute levels of the alkylated DNA damage, DNA deamination and DNA oxidation with good specificity, which are not available using traditional solution-based TdT assays. To the best of our knowledge, no prior study has purposely chosen template-independent polymerases for engineering paper sensors, thus expanding the repertoire of isothermal nucleic acid amplification,^17^ that has been widely used to create various paper-based analytical devices.^2–6^ Moreover, this is the first report of the observation of a pore confinement effect on the catalytic behaviour of polymerases on paper, which encourages us to exploit the high promise of paper-based micro/nanoreactors in biomedicine, biotechnology and biocatalysis. Currently, we are exploring the integration of cell culture, cell lysis, DNA extraction and TdT elongation into a fully integrated paper-based analytical device. We are also integrating this device with a ubiquitous smartphone for image capturing and data processing, which is well suited for ready-to-go testing in low-resource settings. Compared with the commonly used alkaline comet assay and TUNEL assay,^9,18^ this method provides rapid, high throughput, and absolute quantitative measurements of multiple DNA damages induced by exogenous chemical agents. We envision that the method described here will find useful applications in drug development, genotoxicity testing, and environmental toxicology.

## Data availability

Experimental data is available in the ESI† online.

## Author contributions

M. L. devised and developed the project. W. X. conducted most of the experiments. Q. Z. and Y. Y. C. performed some characterization. W. X., J. B., Y. L. and M. L. wrote the paper. All the authors discussed the result.

## Conflicts of interest

There are no conflicts to declare.

## Supplementary Material

SC-013-D1SC04268H-s001
