# Supplementary material for: Quantifying DNA damage on paper sensors via controlled template-independent DNA polymerization
Source: Chem Sci. 2022 Apr 20;13(22):6496–501. doi: 10.1039/d1sc04268h (PMC9172109; doi:10.1039/d1sc04268h)
Supplement: SC-013-D1SC04268H-s001 [file SC-013-D1SC04268H-s001.pdf]

Electronic Supplementary Material (ESI) for Chemical Science.
This journal is © The Royal Society of Chemistry 2022

Section A: Experimental details

A1. Reagents and chemicals

All DNA oligonucleotides (Table S1) were obtained from TaKaRa Bio Inc. (Dalian, China) and purified by 10% denaturing (8 M urea) polyacrylamide gel electrophoresis (dPAGE) or High-Performance Liquid Chromatography (HPLC). TUNEL Apoptosis Detection Kit (FITC) was purchased from YEASEN (Shanghai, China). Human alkyladenine DNA glycosylase (AAG) and AP-endonuclease (APE) were acquired from New England Biolabs (Beijing, China). Phosphate Buffered Saline (PBS), Dulbecco's Modified Eagle Medium (DMEM), Fetal Bovine Serum (FBS), 0.25% Trypsin-EDTA and Fluorescein-12-dUTP (FdU) were obtained from Thermo Scientific (Shanghai, China). *N*7-Methylguanine (7meG) was purchased from Macklin (Shanghai, China). MTT Cell Proliferation and Cytotoxicity Assay Kit was purchased from BBI Life Sciences (Shanghai, China). BCA Protein Assay Kit was purchased from BIODEE (Beijing, China). TIANAmp Genomic DNA Kit was purchased from TIAN GEN (Beijing, China). Filter papers (Grade 1, Grade 2, Grade 3, and Grade 6) with pore sizes ranging from 3 μm to 11 μm were obtained from Whatman. All other chemicals were purchased from Sigma-Aldrich and used without further purification.

A2. Instruments

The fluorescence images were obtained using a Typhoon 5 variable mode imager (GE Healthcare) and analyzed using ImageQuant software (Molecular Dynamics). Paper micro-well plates were printed using a Xerox ColorQube 8560N solid wax printer. Confocal fluorescence imaging was performed on an Olympus FV1000 confocal laser-scanning microscope with a 60× objective lens. Alkylated DNA lesions were analyzed using an Agilent RRLC/6410B Liquid Chromatography-Triple Quadrupole Mass Spectrometer (Agilent, USA). The total DNA concentration was measured using a NanoDrop One Spectrophotometer (ThermoFisher Scientific).

A3. General procedures.

A3.1. TdT elongation of F-pA$_{20}$.

The reaction was performed in 10 μL of 1×TdT reaction buffer (TRB) containing 0.5 μM pA$_{20}$, 20 μM FdU and 35 nM TdT. These reactions were carried out at room temperature (RT) for 5 min, 10 min, 20 min and 30 min before heating at 90 °C for 5 min to deactivate TdT. The resultant products were run on a 20% denaturing polyacrylamide gel electrophoresis (dPAGE) gel (8 M urea) before scanning (Figure 1a, left image). dPAGE analysis of the polymerization products in Figure 2a (left image) was performed in the same way as described above except that different concentrations of pA$_{20}$ (0.5, 1, 2, and 4 μM) were used.

A3.2. TdT elongation of P-pA_{20}.

Wax-printed 96-microzone paper plates (Whatman Grade 1) were prepared using a previously reported wax-printing method,[1] with the diameter of each reaction zone being 4 mm. 2 µL of 1 mg/mL streptavidin was added to each paper well, and incubated at RT for 20 min before washing twice with 20 µL of 1× PBS. After drying at RT, 5 µL of 1 µM biotinylated pA_{20} was added and incubated at RT for 30 min. After washing twice with 20 µL of 1× PBS, 1 µL of 10× TRB, 1 µL of 350 nM TdT, and 2 µL of 100 µM FdU were added (total volume: 10 µL). The reaction was allowed to proceed at RT for 5 min, 10 min, 20 min and 30 min before adding 100 µL of 1×TE buffer to elute the products at 90 °C with shaking for 30 min. The obtained products were concentrated by standard ethanol precipitation and analyzed by 20% dPAGE (Figure 1a, right image). dPAGE analysis of the polymerization products in Figure 2a (right image) was performed in the same way as described above except that different concentrations of pA_{20} (0.5, 1, 2, and 4 µM) were used.

A3.3. Calculation of the average degree of polymerization (*DP*).

We defined the average *DP* as: $DP = TI_{FdU} / TE_{pA20}$, where TI_{FdU} is the total incorporated FdU, and TE_{pA20} is the total elongated pA_{20}. TI_{FdU} of each sample was then determined according to the standard curve of the signal-to-background ratio (S/B) versus FdU concentrations (Figure S4). Because it is difficult to directly quantify TE_{pA20}, we developed a simple strategy that uses a 20-nt DNA molecule with a defined concentration as an internal control (IC), followed by dPAGE and DNA staining with SYBR Gold, a fluorescent DNA binding dye. By determining the fluorescence ratio (FR) of the IC and pA_{20} of each sample, we calculated the concentration of the residual pA_{20} (see below for details), which can be used to estimate TE_{pA20} (Figure S5) based on the total input concentration of pA_{20} (TI_{pA20}).

<u>TdT elongation in solution and on paper (Figure 2b and 2c).</u> For the solution-based strategy, the reaction was performed in 10 µL of 1× TRB containing different concentrations of biotinylated pA_{20} (final concentrations: 0 nM, 0.9 nM, 9 nM, 90 nM and 900 nM), 20 µM FdU and 35 nM TdT. This mixture was incubated at RT for 30 min before heating at 90 °C for 5 min to deactivate the polymerase. 5 µL of the resultant products were added to the streptavidin-coated paper plate. After incubation at RT for 30 min, the paper was washed twice with 20 µL of 1× PBS. The fluorescence intensity of the paper well was scanned. ImageJ software was used to analyze the JPEG images using an 8-bit color scale. The images were then inverted, so that areas that were originally white became black. Based on this scale, an increase in the color resulted in an increase in fluorescence intensity of the well.

For the paper-based strategy, 10 µL of biotinylated pA_{20} (final concentrations: 0 nM, 0.9 nM, 9 nM, 90 nM and 900 nM) was added to the above streptavidin-coated paper well and incubated at RT for 30 min. After washing twice with 20 µL of 1× PBS, 1 µL of 10× TRB, 1 µL of 350 nM TdT,

and 2 μL of 100 μM FdU were added (total volume: 10 μL). The reaction was allowed to proceed at RT for 30 min before adding 5 μL of 0.5 M EDTA to stop the reaction. The paper was washed twice with 20 μL of 1× PBS. The fluorescence intensity of the paper well was scanned and quantified by ImageQuant software.

Analysis of the elongated pA_{20}. For the solution-based strategy, a 5 μL aliquot of the above elongated products was mixed with 5 μL of 2× urea PAGE loading buffer after stopping the solution-based TdT elongation. The above mixture and 5 μL of 400 nM IC were then run on a 20% dPAGE gel, and stained with 1× SYBR Gold at 4 °C for 10 min before scanning. For the paper-based strategy, 100 μL of 1× TE buffer was added to extract the elongated pA_{20} products at 90 °C with shaking for 30 min. The obtained products were concentrated by standard ethanol precipitation and analyzed by 20% dPAGE as described above. The extraction efficiency (η) of the above protocol was determined to be ~88% using fluorophore-labeled pA_{20} (FAM at 3' end, biotin at 5' end).

Calculation of the TE_{pA20}. For the solution-based strategy, the fluorescence intensity of the residual pA_{20} band (F_{pA20}) and the IC band (F_{Ic}) from each sample was estimated using ImageQuant software and used to calculate FR using: $FR = F_{pA20} / F_{Ic}$. From this, we can calculate TE_{pA20} using: $TE_{pA20} = TI_{pA20} - FR \times 2\ pmol \times 2$. Note that 2 pmol was the input concentration of IC, and 2 was the volume correction factor, which was calculated from 10/5 (5 μL of the TdT reaction mixture from 10 μL was used for the analysis of elongated initiators). For the paper-based strategy, TE_{pA20} was calculated using: $TE_{pA20} = TI_{pA20} - FR \times 2\ pmol\ /\ \eta$. Note that η was the DNA extraction efficiency, which was calculated to be ~88%.

Calculation of *DP*. Calculation of *DP* for various DNA initiators including different-sized pA (Figure 2d and 2e), and different sequences (Figure 2f and 2g) was performed in the same way as described above except that the DNA hybridization was first performed in 20 μL of hybridization buffer (50 mM Tris-HCl, pH 7.4 at 25 °C, 100 mM NaCl, 5 mM $MgCl_2$ and 0.02% Tween-20) containing 30 nM biotinylated pA_{20} (or pC_{20}) and 30 nM biotinylated pT_{20} (or pG_{20}). The mixture was heated at 90 °C for 5 min and cooled to RT for 20 min. Calculation of *DP* for elongated P-pA_{20} on different cellulose papers (Figure 3b) was performed in the same way as described above except that Whatman Grade 2 (Φ = 8 μm), Grade 3 (Φ = 6 μm), and Grade 6 (Φ = 3 μm) were used.

A3.4. TdT elongation of S-pA_{20}.

Wax-printed 96-microzone paper plates were prepared on nitrocellulose membranes (Millipore HF120) as described elsewhere,[1] with the diameter of each reaction zone being 4 mm. To facilitate the immobilization of pA_{20} onto the surface, streptavidin was first used to bind the biotinylated pA_{20}. Briefly, 1 nmol of pA_{20} and 30 μL of 1 mg/mL streptavidin were added to 300 μL of 1×PBS buffer. The mixtures were incubated at RT for 2 h before being purified by centrifugation through a 30K membrane (NANOSEP OMEGA, Pall

Incorporation) at 5,000 g for 10 min. The obtained streptavidin-pA_{20} conjugates were washed twice with 100 μL of 1×PBS buffer and collected in ddH_2O. Then 5 μL of the above conjugates were added to each paper well and allowed to dry at RT. After immersion in 1×PBS buffer (containing 10% BSA) for 20 min and washing twice, the obtained bioactive paper was dried at RT. Using this protocol, the streptavidin-pA_{20} conjugates were expected to be immobilized onto the surface of nitrocellulose membrane.[1a]

For surface-based TdT elongation, 1 μL of 10× TRB containing 50 nM pA_{20} conjugates, 1 μL of 350 nM TdT, and 2 μL of 100 μM FdU were added (total volume: 10 μL) to the test zones. The reaction was allowed to proceed at RT for 5 min, 10 min, 20 min and 30 min before adding 100 μL of 1×TE buffer to elute the polymerization products at 90 °C with shaking for 30 min. The obtained products were concentrated by standard ethanol precipitation and analyzed by 20% dPAGE (Figure 3d). Calculation of *DP* for elongated S-pA_{20} (Figure 3e) was performed in the same way as described above except that different concentrations of pA_{20} conjugates (0.9, 9, 90, and 900 nM) were used.

A3.5. Dissociation of cells from primary tissue.

Liver tissue was first cut into 3 to 4 mm pieces with a sterile scissors. After washing twice in PBS buffer, 400 μL of 0.25% trypsin-EDTA was added and incubated at RT for 2 min. Following mixing with 800 μL Dulbecco's Modified Eagle Medium (DMEM), the cell suspension was filtered through a sterile, nylon mesh to separate the dispersed cells and tissue fragments from the larger pieces. The mixture was then centrifuged at 2,000 g at 4 °C for 10 min. The purified cells were re-suspended in DMEM containing 10% fetal bovine serum (FBS), 100 U mL^{-1} of penicillin and 100 μg mL^{-1} of streptomycin for culture.

A3.6. In situ cell imaging.

ZFL cells were exposed to 1 mM DMS at 0 °C for 15 min before being fixed with 4% paraformaldehyde for 20 min. Following incubation with 20 ug/mL protein K in 1× PBS for 10 min, these cells were first treated with 20 U (units) AAG at 37 °C for 15 min, and then incubated with 20 U APE at 37 °C for 60 min. After washing with 1× PBS, these pre-treated cells were mixed with 35 nM TdT and 20 μM FdU in 1× TRB. The mixture was incubated at 37 °C for 60 min, followed by washing and staining with DAPI for 15 min before scanning using a confocal laser scanning microscope (Figure 4b).

A3.7. PAT assay for absolute quantification of 7meG on genomic DNA.

ZFL cells (1× 10^7) were first exposed to 0, 0.5, 0.8, 1, 2, 3, 10 mM DMS for 30 min at 0 °C, and the genomic DNA was then extracted using a DNA extraction kit according to the manufacturer's instructions. 13.5 μg of genomic DNA was mixed with 20 U AAG and 20 U APE in 100 μL of 1× TRB at 37 °C for 60 min. 2 μL of the above mixture was transferred to the paper well. Then 1 μL of 350 nM TdT, and 2 μL of 100 μM FdU were added. The resultant mixture

(total 15 µL) was incubated at RT for 30 min before washing twice with 50 µL of 1× PBS. The fluorescence intensity of the paper well was scanned (Figure 4c) and quantified by ImageQuant software. According to the standard curve of the signal-to-background ratio (S/B) versus FdU concentrations in Figure S2, the total incorporated FdU (T_{FdU}) was determined. Assuming the average *DP* = 3, the total amount of 7meG in 1 ng genomic DNA was then calculated in Figure 4d using: $(T_{FdU} / 3) \times 10^{-12}$ mol × 6.02 × 10^{23}/270 ng.

A3.8. PAT assay for absolute quantification of uracil on genomic DNA.

50 µg of the extracted genomic DNA was first denatured by NaOH (final concentration 0.2 M) for 10 min at 50 °C. Sodium bisulfite and hydroquinone with final concentrations of 62.5/10, 250/10, 1000/10 mM were added to the mixture, and incubated at 50 °C for 3 h under darkness. After adjusting the pH to 8 by NaOH, the pre-treated DNA was desulfonated at RT for 5 min. The genomic DNA was then collected by centrifugation through a 3 K membrane at 5,000 g for 30 min. After washing twice with 100 µL of 1× PBS, 25 µg of genomic DNA was mixed with 15 U UDG and 20 U APE in 100 µL of 1× TRB at 37 °C for 60 min. 2 µL of the above mixture was transferred to the paper well for PAT assay as described in A3.7 to determine the total amount of uracil on genomic DNA.

A3.9. PAT assay for absolute quantification of 3'-OH ends and 8-oxoG on genomic DNA.

30 µg of the extracted genomic DNA was exposed to H_2O_2/Fe^{2+} with final concentrations of 0/0, 1.6/0.3, 6.4/1.2, 25.6/4.8 mM at 37 °C for 2 h. The genomic DNA was collected by centrifugation through a 3 K membrane (NANOSEP OMEGA, Pall Incorporation) at 5,000 g for 30 min. After washing twice by 100 µL of 1× PBS, 10 µg of genomic DNA was resuspended in 100 µL of 1× TRB. 2 µL of the above mixture was transferred to the paper well for PAT assay as described in A3.7 to determine the total amount of 3'-OH ends on genomic DNA. 10 µg aliquot of genomic DNA was mixed with 24 U FPG and 20 U CIAP in 100 µL of 1× TRB at 37 °C for 60 min. 2 µL of the above mixture was transferred to the paper well for PAT assay as described in A3.7 to determine the total amount of 8-oxoG on genomic DNA.

A3.10. Absolute quantification of 7meG, uracil and 8-oxodG by LC-MS.

13.5 µg of the extracted alkylated DNA was first mixed with 20 U AAG in 100 µL of 1× TRB at 37 °C for 60 min. The 7meG was collected by centrifugation through a 3 K membrane (NANOSEP OMEGA, Pall Incorporation) at 5,000 g for 30 min. After washing twice with 100 µL of 1× PBS, the filtrate containing 7meG was collected. 25 µg of the deaminated DNA was first mixed with 20 U FPG in 100 µL of 1× TRB at 37 °C for 60 min. The uracil was collected following the protocol described above. 10 µg of the H_2O_2/Fe^{2+}-treated DNA was mixed with 10 U Nuclease P1, 5 U DNase I, 0.002 U Phosphodiesterase I and 10 U

CIAP in 100 µL of 1× TRB at 37 °C for 60 min. The 8-oxodG was collected following the protocol described above.

Samples containing 7meG, uracil and 8-oxodG were analyzed by QqQ LC/MS2 (Agilent 6410 B) with a XTERRA® MS C18 column (2.1×100 mm, 3.5 µm) at a flow rate of 0.25 mL/min. The temperature of the column was fixed at 40 °C. 5 mM NH_4OAc (solvent A) and CH_3CN (solvent B) were employed as the mobile phase. The gradient elution started with 5% B, increasing to 40% B in 10 min, then held for 2 min. Subsequently, it returned to initial conditions (5% B) in 0.01 min, then was held for 7 min for column back-conditioning. The mass spectrometry detection was performed using the positive electrospray ionization mode. The 7meG was monitored by multiple reaction monitoring (MRM) using mass transition (precursor ions→ product ions, m/z 166.0 → m/z 149.0) for quantification, (precursor ions→ product ions, m/z 166.0 → m/z 124.0) for confirmation. The uracil was monitored by multiple reaction monitoring (MRM) using mass transition (precursor ions→ product ions, m/z 113.0 → m/z 70.0) for quantification, (precursor ions→ product ions, m/z 113.0 → m/z 96.0) for confirmation. The 8-oxodG was monitored by multiple reaction monitoring (MRM) using mass transition (precursor ions→ product ions, m/z 284.0 → m/z 168.0) for quantification, (precursor ions→ product ions, m/z 284.0 → m/z 140.0) for confirmation. The ion source parameters were as follows: gas temperature, 350 °C; gas flow, 8 L/min; nebulizer, 25 psi.

Reference

1. (*a*) M. Liu, C. Y. Hui, Q. Zhang, J. Gu, B. Kannan, S. Jahanshahi-Anbuhi, C. D. M. Filipe, J. D. Brennan and Y. Li, *Angew. Chem. Int. Ed.,* 2016, **55**, 2709; (*b*) C. Y. Hui, M. Liu, Y. Li and J. D. Brennan, *Angew. Chem. Int. Ed.,* 2018, **57**, 4549; (*c*) M. Liu, Q. Zhang, B. Kannan, G. A. Botton, J. Yang, L. Soleymani, J. D. Brennan and Y. Li, *Angew. Chem. Int. Ed.,* 2018, **57**, 12440.

Section B: Supporting Tables and Figures

Table S1. Sequences of DNA oligonucleotides used in this work

Name	Sequence (5′-3′)
pA_{10}	AAAAA AAAAA (5’ biotin)
pA_{20}	AAAAA AAAAA AAAAA AAAAA (5’ biotin)
pA_{30}	AAAAA AAAAA AAAAA AAAAA AAAAA AAAAA (5’ biotin)
pA_{40}	AAAAA AAAAA AAAAA AAAAA AAAAA AAAAA AAAAA AAAAA (5’ biotin)
pT_{20}	TTTTT TTTTT TTTTT TTTTT (5’ biotin)
pG_{20}	GGGGG GGGGG GGGGG GGGGG (5’ biotin)
pC_{20}	CCCCC CCCCC CCCCC CCCCC (5’ biotin)
uracil-containing pA_{19}	AAAAA AAAAA UAAAA AAAAA AAAAA AAAAA (5’ biotin, 3’ PO_4)
DNA pool	NNNNN NNNNN NNNNN NNNNN NNNNN NNNNN NNNNN NNNNN (5’ biotin, N = A, G, C, or T)
Internal control (IC)	AAAAA AAAAA AAAAA AAAAA
fluorophore-labeled pA_{20}	AAAAA AAAAA AAAAA AAAAA-FAM (5’ biotin)

Table S2. The proportion of major products on paper

Products	Repeat				Coefficient of variation (CV)[a]
	1	2	3	4	
pA_{21}	59%	62%	52%	56%	7.5%
pA_{22}	26%	22%	21%	25%	10.1%
pA_{23}	12.5%	13%	14%	16%	11.1%
pA_{24}	2.5%	3%	3%	3%	8.7%

[a]CV% = (standard deviation / average)%.

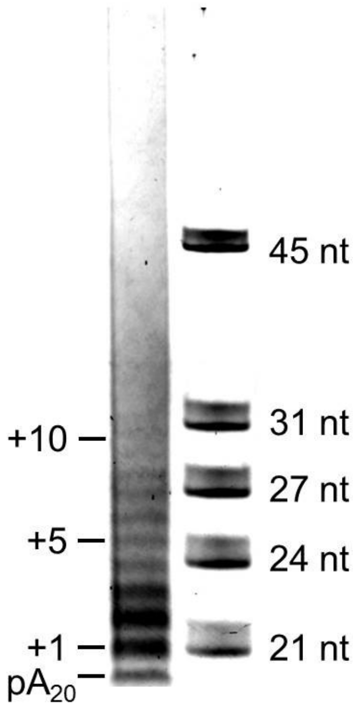

Fig. S1. 20% dPAGE analysis of the polymerization products by TdT elongation. The products were stained with 1× SYBR Gold at 4 °C for 10 min before scanning. DNA ladders ranging from to 21 nt to 45 nt are provided. nt: nucleotide.

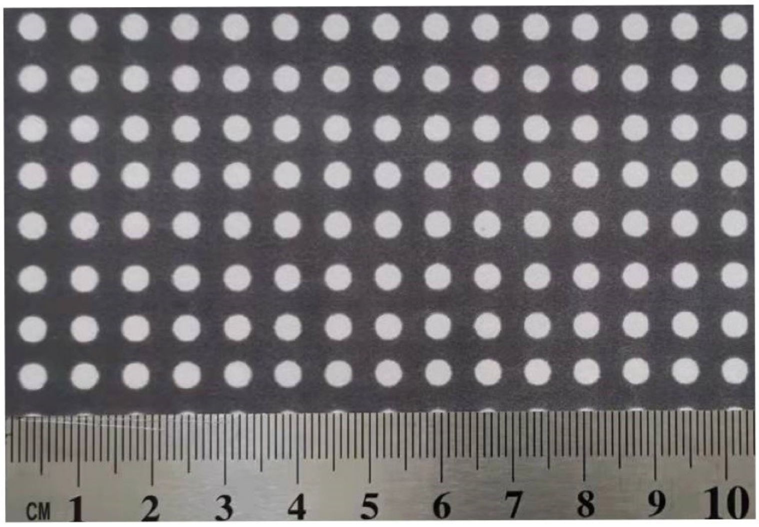

Fig. S2. Image of a Grade 1 paper plate.

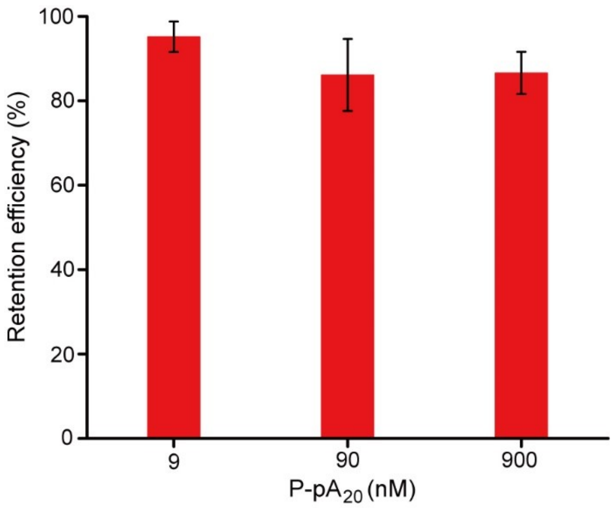

Fig. S3. Retention efficiencies for P-pA_{20} at various concentrations.

Experimental details: 10 μL of biotinylated pA_{20} (FAM at 3' end) with final concentrations of 9, 90, and 900 nM was added to the streptavidin-coated paper well and incubated at RT for 30 min. and incubated at RT for 30 min. The paper was washed twice with 20 μL of 1× PBS. The fluorescence intensity of the paper well was scanned and quantified by ImageQuant software.

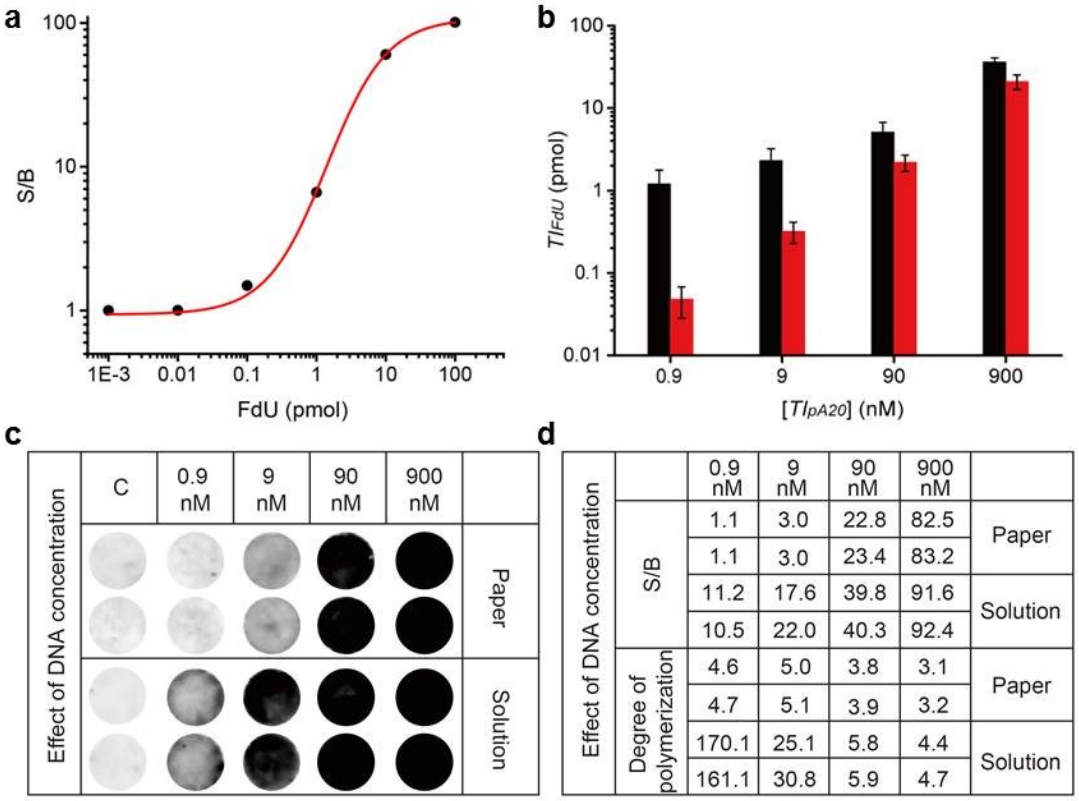

Fig. S4. a) Standard curve of signal-to-background ratio (S/B) vs. FdU amounts (pmol) on cellulose paper. b) The calculated TI_{FdU} for TdT elongation in solution (black column) and on cellulose paper (red column) at various TI_{pA20}. c) Fluorescence images and d) data analysis of the elongated pA_{20} under different input concentrations. For solution-based strategy, the reaction mixtures were transferred onto paper plate before scanning. C: control.

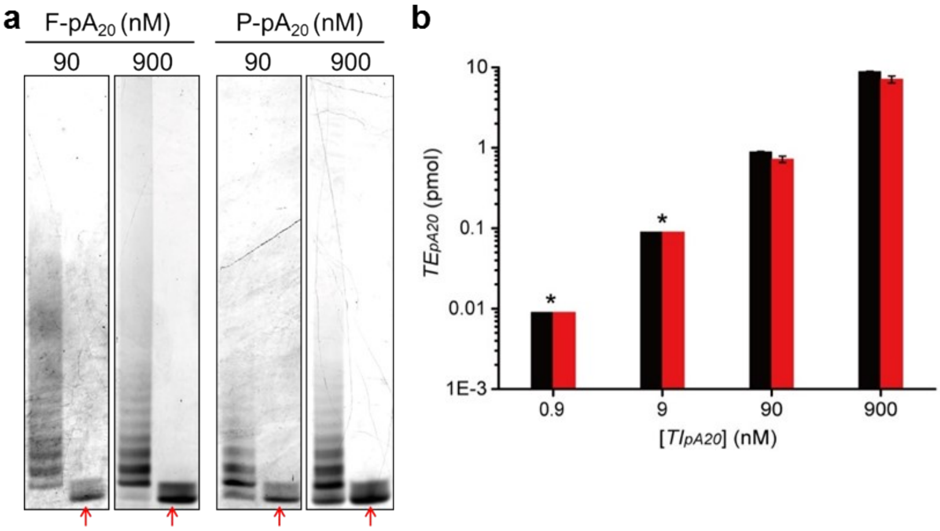

Fig. S5. dPAGE analysis of the products synthesized by TdT at high concentrations of pA20. The red arrow indicates the internal control (IC) band (20 nt), and the gel was stained with 1× SYBR Gold before scanning. b) The calculated TE_{pA20} for TdT elongation in solution (black column) and on cellulose paper (red column) at various TI_{pA20}. *pA_{20} with low input amounts cannot be resolved on 20% dPAGE gel. The ratio of elongated pA_{20} was considered to be 100% when low concentrations of pA_{20} were tested. The error bars represent standard deviations of three independent experiments.

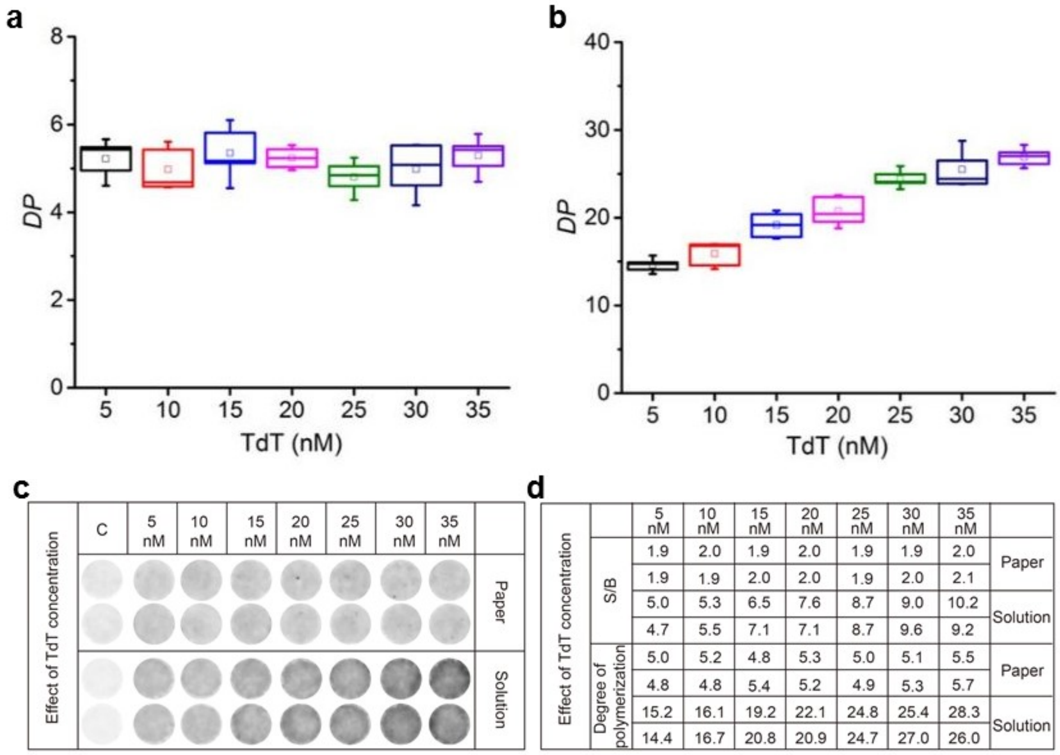

Effect of TdT concentration		5 nM	10 nM	15 nM	20 nM	25 nM	30 nM	35 nM	
	S/B	1.9	2.0	1.9	2.0	1.9	1.9	2.0	Paper
		1.9	1.9	2.0	2.0	1.9	2.0	2.1	
		5.0	5.3	6.5	7.6	8.7	9.0	10.2	Solution
		4.7	5.5	7.1	7.1	8.7	9.6	9.2	
	Degree of polymerization	5.0	5.2	4.8	5.3	5.0	5.1	5.5	Paper
		4.8	4.8	5.4	5.2	4.9	5.3	5.7	
		15.2	16.1	19.2	22.1	24.8	25.4	28.3	Solution
		14.4	16.7	20.8	20.9	24.7	27.0	26.0	

Fig. S6. Effect of TdT concentration on the average *DP* for a) paper- and b) solution-based strategy. Reactions were carried out with 20 μM FdU, 5 nM pA_{20} initiator and varying concentrations of TdT at room temperature for 30 min. c) Fluorescence images and d) data analysis of the elongated pA_{20} under different TdT concentrations. For solution-based strategy, the reaction mixtures were transferred onto paper plate before scanning. C: control.

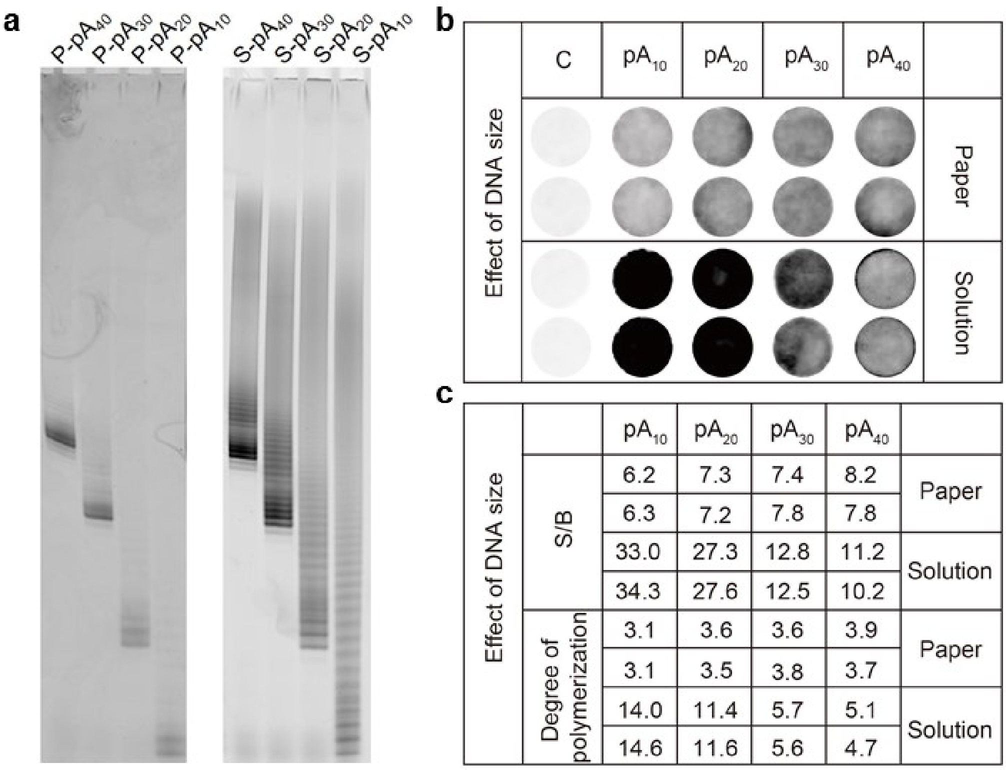

Effect of DNA size		pA10	pA20	pA30	pA40	
	S/B	6.2	7.3	7.4	8.2	Paper
		6.3	7.2	7.8	7.8	
		33.0	27.3	12.8	11.2	Solution
		34.3	27.6	12.5	10.2	
	Degree of polymerization	3.1	3.6	3.6	3.9	Paper
		3.1	3.5	3.8	3.7	
		14.0	11.4	5.7	5.1	Solution
		14.6	11.6	5.6	4.7	

Fig. S7. a) TdT elongation of different-sized pA initiator on paper (left image) and in solution (right image). Reactions were carried out with 20 μM FdU, 30 nM pA initiator and 35 nM TdT at RT. Aliquots of the reactions taken at 30 min were analyzed on a 20% dPAGE (8 M urea) gel. b) Fluorescence images and c) data analysis of the elongated pA_{10}, pA_{20}, pA_{30} and pA_{40}. For solution-based strategy, the reaction mixtures were transferred onto paper plate before scanning. C: control.

a

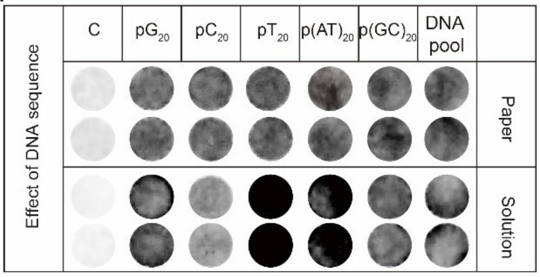

b

Effect of DNA sequence

	pG_{20}	pC_{20}	pT_{20}	$p(AT)_{20}$	$p(GC)_{20}$	DNA pool	
S/B	3.9	3.9	4.4	4.6	4.4	3.8	Paper
	3.8	3.8	4.3	4.1	4.6	4.0	
	12.9	5.8	19.1	14.9	8.2	9.4	Solution
	13.4	6.1	18.6	15.7	7.3	9.0	
Degree of polymerization	3.3	3.3	3.7	3.8	3.7	3.2	Paper
	3.2	3.2	3.6	3.5	3.9	3.4	
	9.5	4.8	13.3	10.8	6.5	7.3	Solution
	9.8	5.0	13.0	11.3	5.8	7.0	

Fig. S8. a) Fluorescence images and b) data analysis of the elongated pG_{20}, pC_{20}, pT_{20}, $p(AT)_{20}$, $p(GC)_{20}$ and DNA pool. For solution-based strategy, the reaction mixtures were transferred onto paper plate before scanning. C: control.

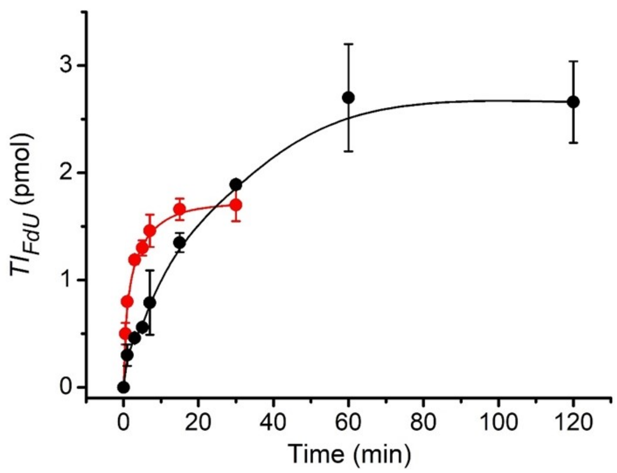

Fig. S9. Total incorporated FdU as a function of time. The reaction rates of F-pA_{20} and P-pA_{20} are compared.

Experimental details: For the solution-based strategy, the reaction was performed in 10 μL of 1× TRB containing 50 nM pA_{20}, 20 μM FdU and 35 nM TdT. This mixture was incubated at 30 °C for 1, 3, 5, 10, 20, 30, 60 and 120 min before heating at 90 °C for 5 min to deactivate the polymerase. 10 μL of the resultant products were added to the streptavidin-coated paper plate. After incubation at RT for 30 min, the paper was washed twice with 20 μL of 1× PBS. The fluorescence intensity of the paper well was scanned and quantified by ImageQuant software.

For the paper-based strategy, 10 μL of 50 nM pA_{20} was added to the streptavidin-coated paper well and incubated at RT for 30 min. After washing twice with 20 μL of 1× PBS, 1 μL of 10× TRB, 1 μL of 350 nM TdT, and 2 μL of 100 μM FdU were added (total volume: 10 μL). The reaction was allowed to proceed at RT for 1, 3, 5, 10, 20, 30 min before adding 5 μL of 0.5 M EDTA to stop the reaction. The paper was washed twice with 20 μL of 1× PBS. The fluorescence intensity of the paper well was then scanned and quantified by ImageQuant software.

According to the standard curve of the signal-to-background ratio (S/B) versus FdU concentrations in Figure S3, the TI_{FdU} was determined.

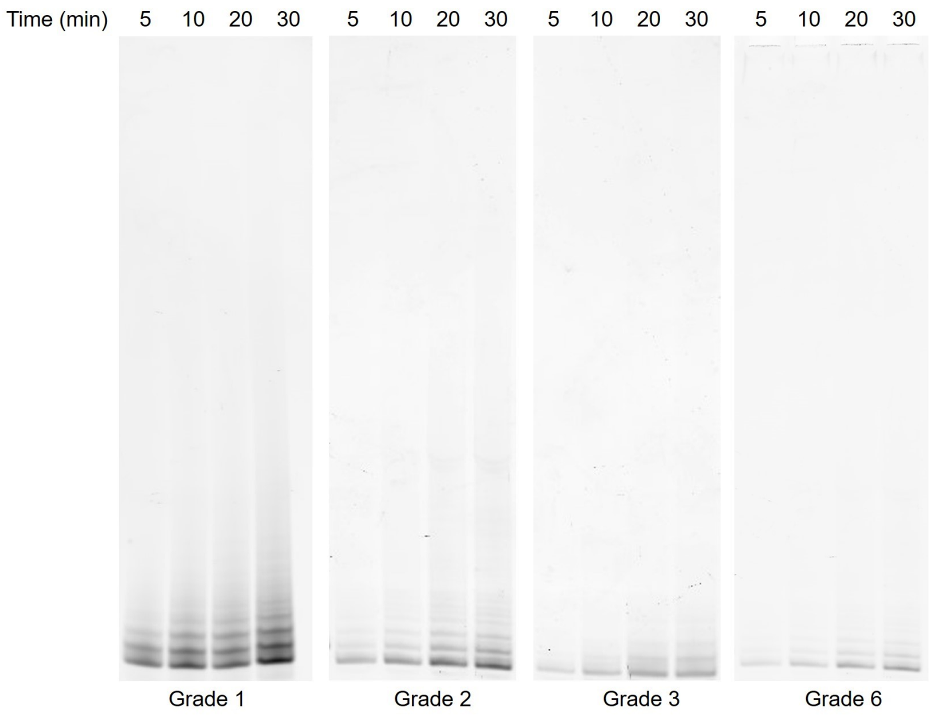

Fig. S10. TdT elongation of pA_{20} on different cellulose papers.

a

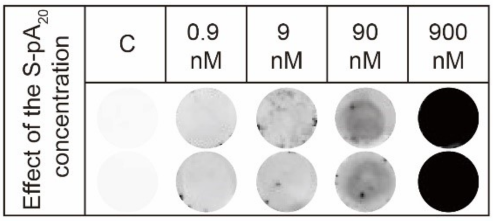

b

Effect of the S-pA_{20} concentration

		0.9 nM	9 nM	90 nM	900 nM
S/B		7.2	8.9	17.8	77.4
		6.1	8.6	16.2	75.5
Degree of polymerization		117.5	14.1	2.5	2.1
		102.0	13.6	2.3	1.9

Fig. S11. a) Fluorescence images and b) data analysis of the elongated pA_{20} on the surface of nitrocellulose membrane. C: control.

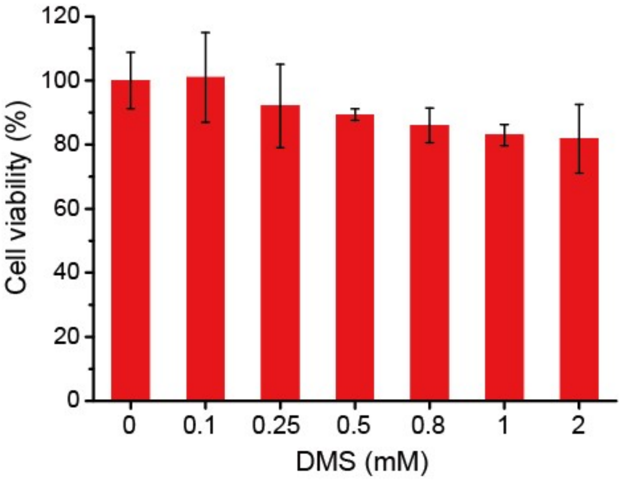

Fig. S12. Cellular viability of ZFL cells exposed to DMS at various concentrations (0-2 mM).

Experimental details: ZFL cells (1 × 10^4 cells/well) were cultured in 96-well microtiter plates and incubated at 37 °C in 5% CO_2 for 6 h. The original medium was discarded, and new media containing different concentrations of DMS was added. After incubating at 0 °C for 30 min, the medium was removed. 90 μL of fresh medium and 10 μL of MTT solution were added and incubated at 37 °C, in a 5% CO_2 incubator for 4 h. Cell viability was then determined according to the manufacturer's instructions.

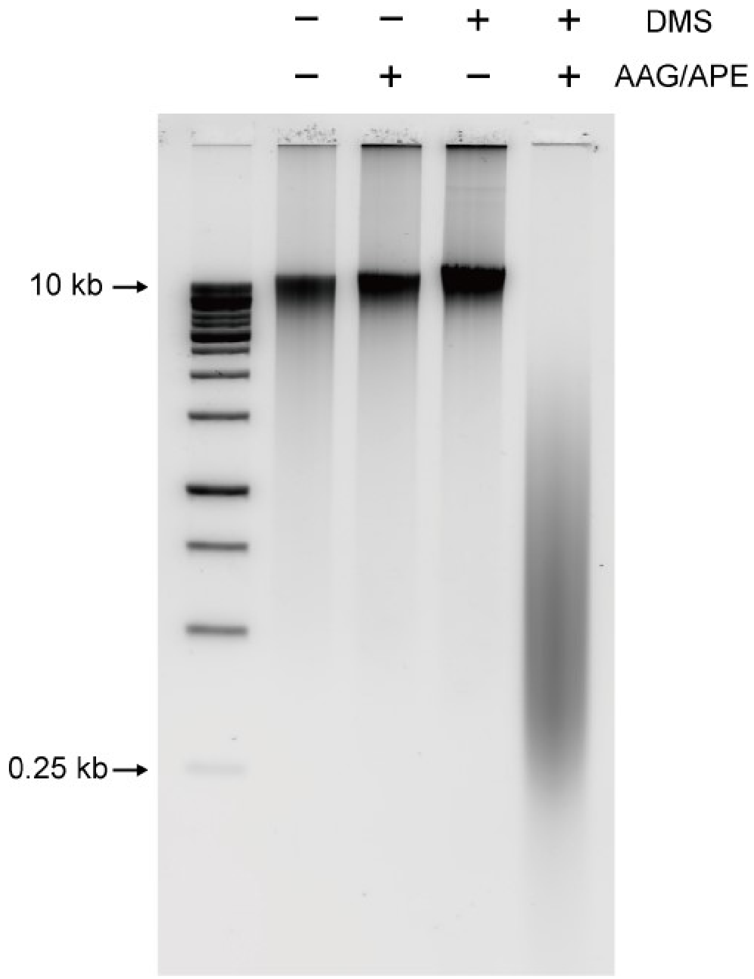

Fig. S13. Agarose gel electrophoresis analysis of the genomic DNA. ZFL cells (1×10^7) were first exposed to 1 mM DMS for 30 min at 0 °C, and the genomic DNA was then extracted using a DNA extraction kit according to the manufacturer's instructions. 13.5 μg of genomic DNA was mixed with 20 U AAG and 20 U APE in 100 μL of 1× TRB at 37 °C for 60 min. Various control reactions lacking DMS, AAG/APE, or a combination of these were also set up in the same way. The genomic DNA (~ 200 ng) from these reactions were analyzed by 2% agarose gel electrophoresis.

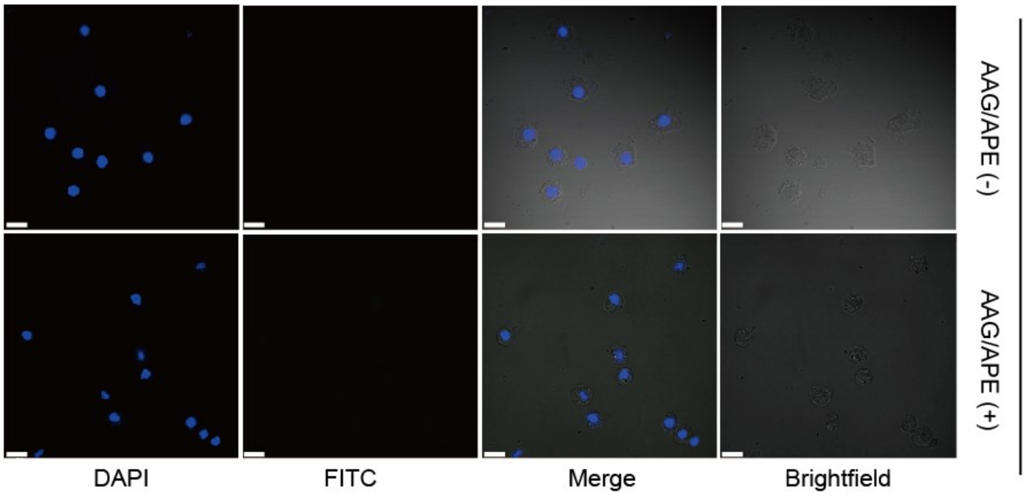

Fig. S14. In situ labeling of 7meG in normal ZFL cells using AAG/APE-mediated TUNEL assay. Scale bar: 10 μm.

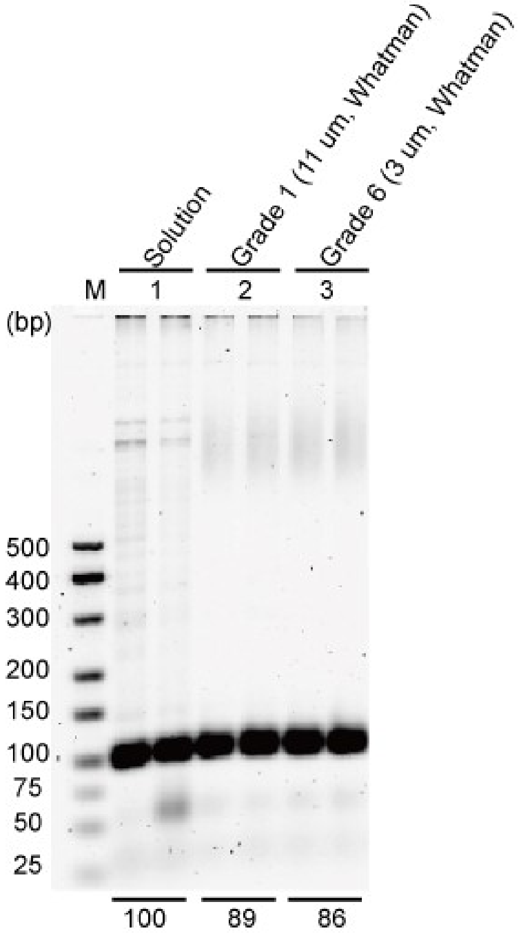

Fig. S15. Characterization of the DNA capture capabilities of two different cellulose papers using PCR.

Experimental details: 150 ng of genomic DNA was pipetted onto the paper sensor and incubated at RT for 30 min before washing twice with 50 μL of 1× PBS. The filter paper was cut off and transferred to a tube for PCR. The sequences of primers were as follows:
forward primer: 5'-GGCCCATCCATCGTTCACAG-3';
reverse primer: 5'-CGAGAGTTTAGGTTGGTCGTTCG-3'.
Thermal cycles were typically performed as follows: 95 °C for 3 min; 30 cycles of 94 °C for 30 s, 58 °C for 30 s and 72 °C for 45 s; 72 °C for 10 min. An agarose gel (2%) electrophoresis was performed to check the product.

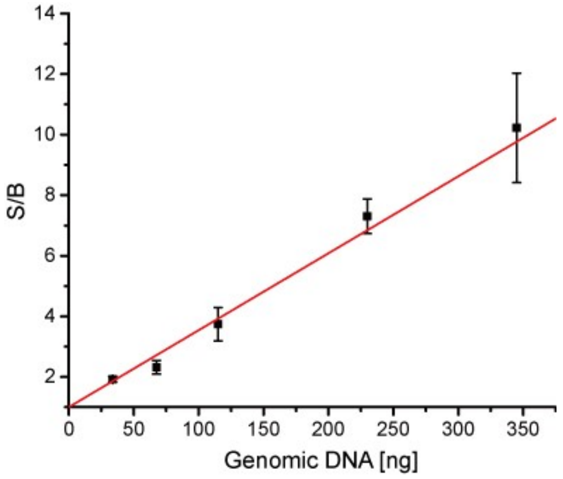

Fig. S16. Detection sensitivity of PAT assay for quantification of DNA alkylation damage.

Experimental details: 17.25 μg of genomic DNA was mixed with 20 U AAG and 20 U APE in 100 μL of 1× TRB at 37 °C for 60 min. 2 μL of the above mixture containing 33.75, 67.5, 115, 230, and 345 ng DNA was transferred to the paper well. Then 1 μL of 350 nM TdT, and 2 μL of 100 μM FdU were added. The resultant mixture (total 15 μL) was incubated at RT for 30 min before washing twice with 50 μL of 1× PBS. The fluorescence intensity of the paper well was scanned and quantified by ImageQuant software. Based on the basis of the 3σ/slope (σ, standard deviation of the blank samples), the detection sensitivity of this assay was 7.56 ng genomic DNA. Thus the detection limit of 7meG was calculated using: 7.56 ng × 13.5 × 10^8 per ng DNA = 10^{10} molecules.

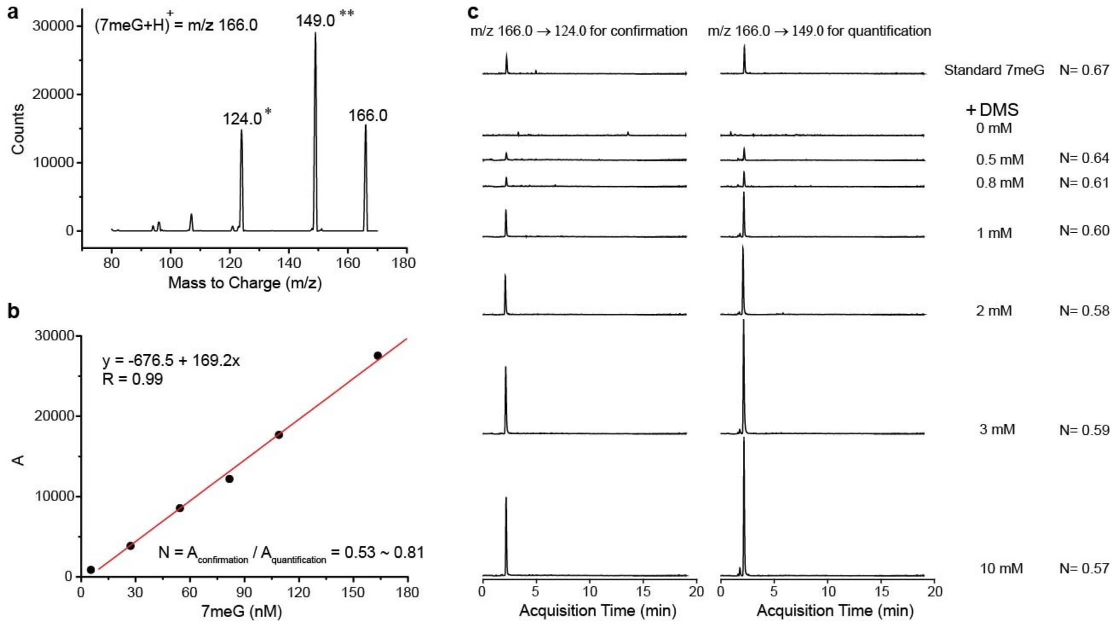

Fig. S17. Analysis of 7meG on genomic DNA. a) Product ion spectrum of 7meG by LC-MS. b) Calibration curves for the LC/MS^2 measurement of 7meG. c) MRM chromatogram of 7meG in ZFL cells pre-treated with different concentrations of DMS. The 7meG was monitored by multiple reaction monitoring (MRM) using mass transition (precursor ions→ product ions, m/z 166.0 → m/z 149.0) for quantification, (precursor ions→ product ions, m/z 166.0 → m/z 124.0) for confirmation.

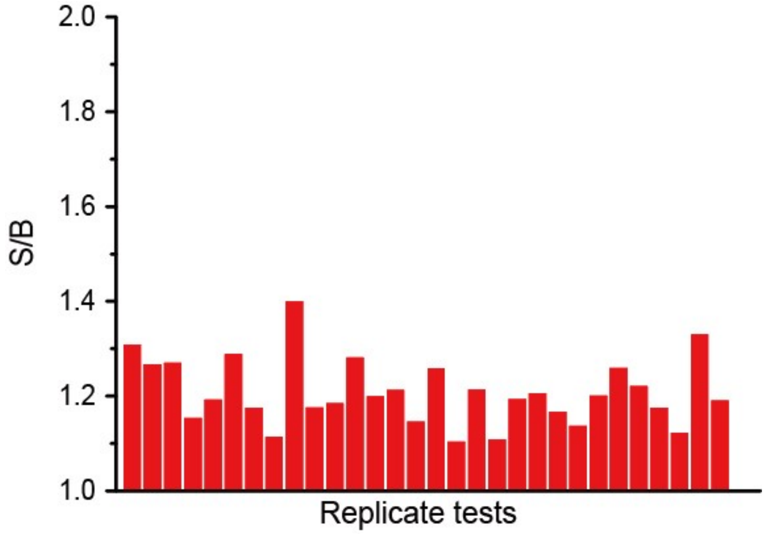

Fig. S18. S/B values for 30 individual paper sensors in the detection of 7meG. ZFL cells (1× 10^7) were first treated with 0.3 mM DMS for 30 min before lysis.

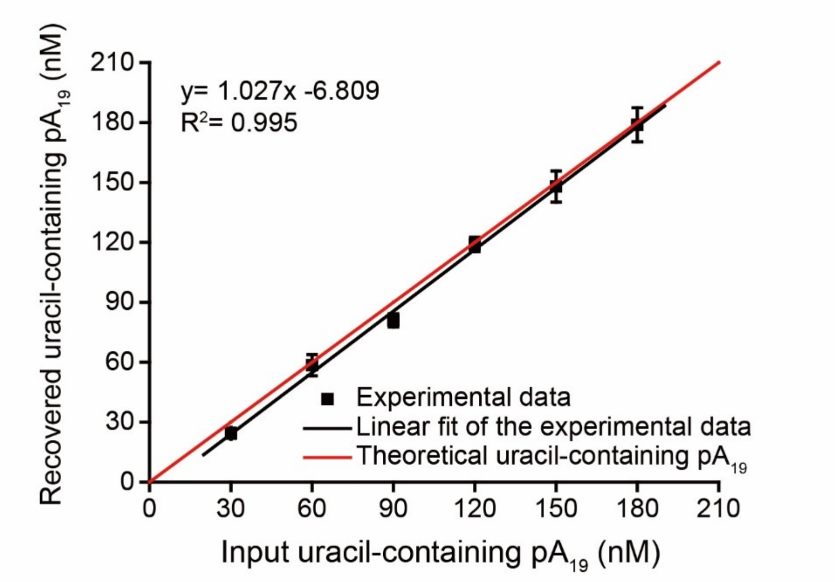

Fig. S19. Quantification of uracil-containing pA_{19} using UDG/APE-mediated PAT assay. Percentage values indicate recovery yields. The error bars represent standard deviations of three independent experiments.

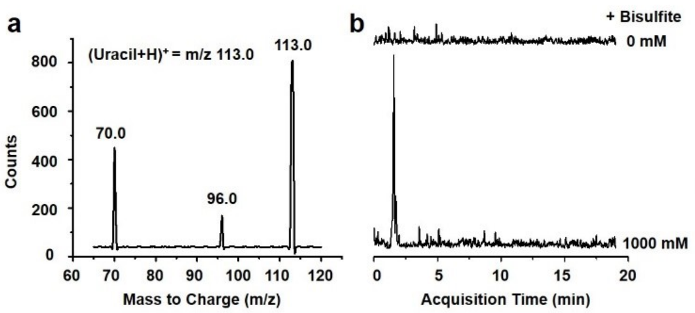

Fig. S20. Analysis of uracil on genomic DNA. a) Product ion spectrum of uracil by LC-MS. b) MRM chromatogram of uracil in ZFL cells pre-treated with bisulfite. c) Absolute quantification of uracil by mass spectrometric method and PAT assay. The uracil was monitored by multiple reaction monitoring (MRM) using mass transition (precursor ions→ product ions, m/z 113.0 → m/z 70.0) for quantification, (precursor ions→ product ions, m/z 113.0 → m/z 96.0) for confirmation.

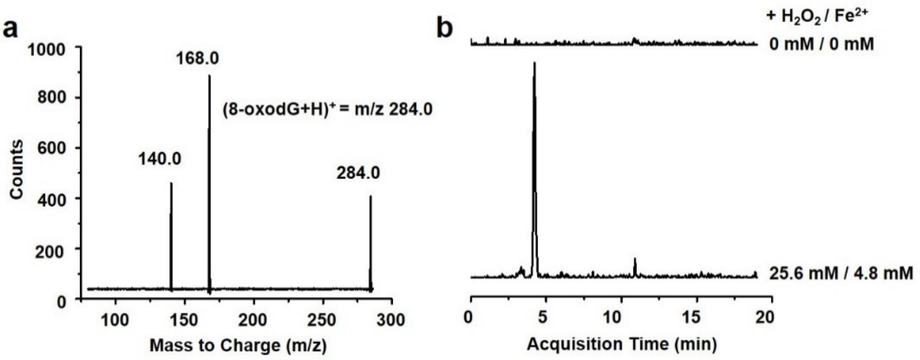

Fig. S21. Analysis of 8-oxodG on genomic DNA. a) Product ion spectrum of 8-oxodG by LC-MS. b) MRM chromatogram of 8-oxodG in ZFL cells pre-treated with Fenton reagents (H_2O_2/Fe^{2+}). The 8-oxodG was monitored by multiple reaction monitoring (MRM) using mass transition (precursor ions→ product ions, m/z 284.0 → m/z 168.0) for quantification, (precursor ions→ product ions, m/z 284.0 → m/z 140.0) for confirmation.
